# Artificial Tactile Sensing Neuron with Tactile Sensing Ability Based on a Chitosan Memristor

**DOI:** 10.1002/advs.202308610

**Published:** 2024-03-14

**Authors:** Lu Wang, Peng Zhang, Zhiqiang Gao, Dianzhong Wen

**Affiliations:** ^1^ School of Electronic Engineering Heilongjiang University Harbin 150080 China

**Keywords:** data processing, information storage, memristor, touch‐sensing neuron

## Abstract

Owing to the highly parallel network structure of the biological neural network and its triggered processing mode, tactile sensory neurons can realize the perception of external signals and the functions of perception, memory, and data processing by adjusting the synaptic weight. In this paper, a piezoresistive pressure sensor is combined with a memristor to design an artificial tactile sensory neuron. The polyurethane sponge sensor has excellent sensitivity and can convert physical stimuli into electrical signals, and the chitosan‐based memristor has stable bipolar resistive switching characteristics, allowing further information to be memorized and processed. The neuron can respond to tactile stimuli of different degrees, durations, and frequencies; realize potentiation/depression modulation, paired‐pulse facilitation, and spike‐timing‐dependent plasticity; exhibit spike‐rate‐dependent plasticity; and store and erase tactile information through memistor state switching, which has great application potential in biological sensing systems.

## Graphical abstract

A piezoresistive pressure sensor is combined with a memristor to design an artificial tactile sensory neuron. The neuron can respond to tactile stimuli of different degrees, durations and frequencies; realize potentiation/depression modulation, paired‐pulse facilitation, and spike‐timing‐dependent plasticity; exhibit spike‐rate‐dependent plasticity; and store and erase tactile information through memistor state switching, which has great application potential in biological sensing systems.

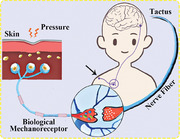

## 1 Introduction

Perception is the interface between artificial systems and the real world, and it is also the basic function required by intelligent artificial systems.^[^ ^1^ ^]^ With the continuous development of information technologies such as artificial intelligence,^[^ ^2^ ^]^ big data, and 5G,^[^ ^3^ ^]^ major changes have taken place in the fields of human‐computer interaction, intelligent perception systems,^[^ ^4^ ^]^ and bionic robots.^[^ ^5^ ^]^ The perception of tactile information relies on integrated perception, refinement, and learning activities that greatly influence our interactions with the environment.^[^ ^6^ ^]^ Tactile sensors show great potential in areas such as electronic skin,^[^ ^7^ ^]^ wearable electronics,^[^ ^8^ ^]^ intelligent robotics,^[^ ^9^ ^]^ human‐computer interaction,^[^ ^10^ ^]^ and artificial touch.^[^ ^11^ ^]^ To enable perceptual learning in robots and prosthetics, it is necessary to create next‐generation, low‐power, flexible, and perceptually learning artificial sensory neurons.^[^ ^12^ ^]^

Compared with traditional electronic devices, biological sensory organs can detect and process external information and transmit the processed signals to the brain for final information judgment.^[^ ^13^ ^]^ The biological perceptual system has these advantages because synapses play a crucial role in the system.^[^ ^14^, ^15^ ^]^ Signal processing, memory, learning, and forgetting are achieved by adjusting the weight of synapses, which is the basis for biological perception and information processing.^[^ ^16^ ^]^ Different from traditional sensing information processing methods, biomimetic neuromorphic sensing systems based on artificial synaptic devices mix analog signals and digital signals and can realize self‐adaptation,^[^ ^17^, ^18^, ^19^ ^]^ self‐organization, self‐learning, and other functions on multiple spatial and temporal scales, which greatly reduces the power consumption of the system.^[^ ^20^ ^]^ As an important component of biomimetic neuromorphological perception systems, biomimetic artificial synaptic devices have attracted widespread attention in recent years due to their special processor/memory configuration structure, which can modulate and process a large amount of nonstandard data.^[^ ^21^ ^]^ Memristor devices have low power consumption, high running speed, and good storage capacity.^[^ ^22^, ^23^ ^]^ When TiO_2_, MoO_3_, CeO_2_, and other inorganic oxides are combined with complementary metal oxide semiconductor technology as active layers, memristors with excellent memory function can be produced,^[^ ^24^, ^25^, ^26^ ^]^ but the process is relatively complex, and a large amount of electronic waste will be generated as the device's service life is exhausted.^[^ ^27^ ^]^ Because of their excellent biocompatibility, degradability and low cost, biomaterials are widely used in the production of memristors,^[^ ^28^ ^]^ which can also realize resistance switching behavior and memory storage function.^[^ ^29^ ^]^ In recent years, biomemristor devices based on lotus leaves,^[^ ^30^ ^]^ egg whites,^[^ ^31^ ^]^ starch,^[^ ^32^ ^]^ carrageenan,^[^ ^33^ ^]^ etc., have been reported to exhibit a higher ON/OFF ratio and better cycling endurance.^[^ ^34^ ^]^ Zhang et al. demonstrated an artificial touch neuron system that mimics biological sensory neurons by combining piezoresistive sensors and memristors to integrate time‐dependent tactile stimuli for sensory processing and learning.^[^ ^35^ ^]^ Lee et al. proposed a simple threshold switch synaptic device to simulate synapses that exhibit both resistance switching behavior and tunable synaptic weight behavior.^[^ ^36^ ^]^

In this paper, the active layer of a chitosan （CS）‐based memristor was doped with multi‐walled carbon nanotubes (MWCNTs) to realize an adjustable switching ratio of the device to match the fabricated sensor. The device showed good cycling stability and retention characteristics. The gauge factor (GF) of the flexible tactile sensor made of polyurethane sponge is 75.4 within the 0–50% strain range and 1067.2 within the 50–74% strain range, which met the need for tactile information acquisition. An artificial touch neuron composed of two devices can realize the storage and erasure of tactile information. By applying cyclic stress, neurons are able to achieve enhanced/suppressed synaptic weight modulation, double pulse facilitation, pulse number and rate‐dependent plasticity. These neurons can integrate time‐related tactile stimuli for sensory information processing and learning.

## 2 Results and Discussion

### 2.1 CS Memristor

**Figure 1:**
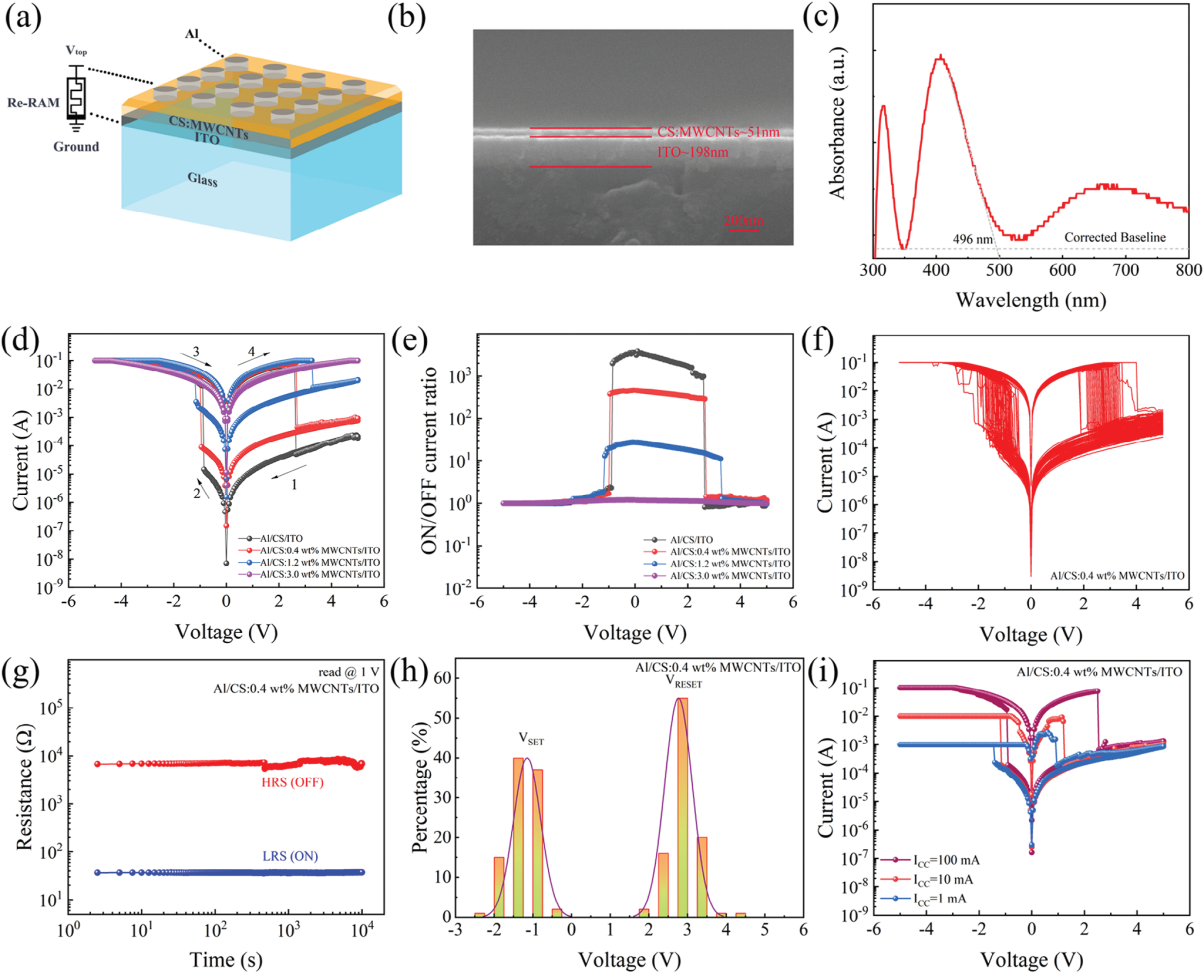
a) Structure of the memristor. b) SEM image of the section of the memristor. c) UV spectrum of MWCNTs. d) *I*–*V* curves for devices. e) Current on/off ratio. f) Repeated test. g) Holding time test. h) Threshold voltage distribution statistics. i) Multistage storage.

**Figure** **1**a shows the device structure. The active layer of MWCNTs is ≈45 nm, and the active layer of indium tin oxide (ITO) is ≈220 nm (Figure 1b). The ultraviolet spectrum of the MWCNTs is shown in Figure 1c, and the absorption wavelength of the MWCNTs was 496 nm. From equation *Eg*  = *hc*/λ, it follows that the bandgap width *Eg* is ≈2.5 eV, where *h* is Planck's constant and *c* is the speed of light.

To study the effects of MWCNTs at different concentrations on the electrical characteristics of memristors, the electrical characteristics of the Al/CS:MWCNTs/ITO devices were tested at room temperature. The observed current‒voltage relationship showed bipolar resistance switching characteristics. A forward voltage is applied to the top electrode of the memristor, and the bottom electrode of the ITO is grounded. The device was converted from the HRS to the LRS when the bias voltage applied to the Al/CS:0.4 wt.% MWCNTs/ITO device reached V_SET_ (−1 V). During the voltage sweep from 0 to 5 V, the device was converted from the LRS to the HRS when the bias voltage was applied up to the reset voltage V_RESET_ (2.7 Al/CS/ITO and Al/CS: The V_SET_ and V_RESET_ of the 1.2 wt.% MWCNTs/ITO devices are −0.9 and 2.6 and −1.2 and 3.3 V, respectively. Figure 1d shows the bipolar switching behavior of the Al/CS:MWCNTs/ITO devices with different concentrations of MWCNTs. As the concentration of MWCNTs increases, the switching current ratio of the devices decreases. The Al/CS/ITO and Al/CS:0.4, 1.2, and 3 wt.% switching current ratios of the MWCNTs/ITO devices were 2.55 × 10,3 3.95 × 10,2 22.7, and 1, respectively (Figure 1e). To study the stability of the memristor, repeated *I*–*V* scanning was performed on the same cell of the device at room temperature (Figure 1f). Al/CS:MWCNTs/ITO devices could achieve more than 100 *I*‒*V* scans, and the size of the switching ratio window was basically unchanged. The repeatability test results for the Al/CS/ITO device and Al/CS (1.2 and 3 wt.%) MWCNTs/ITO devices are shown in Figure S1 (Supporting Information).

Figure 1g shows the data retention test results for the Al/CS/ITO and Al/CS:MWCNTs/ITO devices at room temperature (with a DC bias of 1 V). The resistance values of the LRS and HRS devices remained stable for 104 s without significant change, which reflects the strong retention capability of the devices. Figure 1h Repeated *I*‒*V* test of the device and statistical analysis of the V_SET_ and V_RESET_ devices. The results showed that the V_SET_ and V_RESET_ of the biomemristor based on CS and CS:MWCNTs nanocomposites were relatively concentrated. *I*–*V* scanning of the Al/CS:MWCNTs/ITO devices was performed by adjusting the limiting current in the negative voltage region. The results showed that the devices could achieve multivalue storage. Figure 1i shows the *I*–*V* curve of the Al/CS:0.4 wt.% MWCNTs/ITO device after the limiting current in the negative voltage scanning area is set to 100, 10, and 1 mA. The change in the limiting current has almost no effect on the R_HRS_ of the device, while the change in the R_LRS_ is more obvious. The different reset behaviors of the device in the positive voltage region indicate that the positioning information of the device in the negative voltage region has been successfully stored in the device.

**Figure 2:**
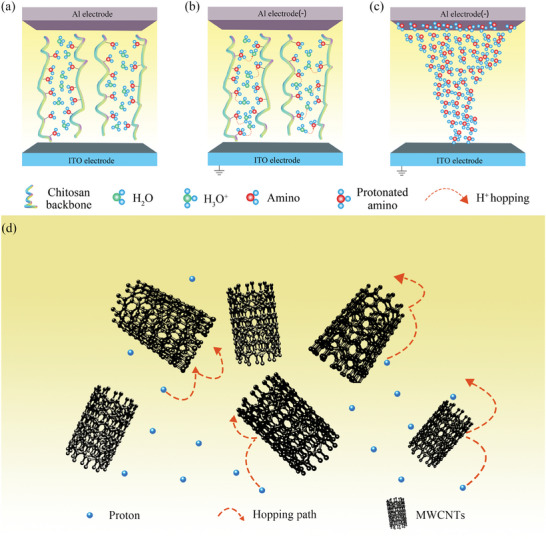
a) Particle distribution in the initial state of the device. b) Particle movement when a negative voltage is applied at the top electrode. c) Conductive filament formation. d) Proton movement through carbon nanotubes.

In Al/CS/ITO devices, the inert metal aluminum is used as the top electrode, there is basically no ionization when forward bias is applied, and the conductive filaments that form during device conduction are not related to the metal wire. CS is a linear polymer with a chain‐like molecular structure. When a solution of CS acetic acid was prepared, proton acid was doped on the CS molecules. The CS molecules first adsorb acid ions and become proton conductors. With increasing protonation, CS gradually dissolves in water. CS chains can form CS films containing hydrogen bonds and water molecules through self‐assembly and aggregation via hydrogen bond interactions and hydrophobic interactions.^[^ ^37^ ^]^ Protected amino acids with a positive charge are an important source of internal conduction for CS molecules. When the number of free cations in the CS chain is the highest, the maximum conductivity will be reached, and the device will be transformed from an HRS to an LRS. In the initial state, a small amount of protonated amino groups exist on the CS molecular chain in the active layer of the device, and water molecules and hydronium ions are randomly distributed in the active layer (**Figure** **2**a).

When a negative voltage is applied to the top electrode, more hydrogen ions are ionized by water molecules under the action of an electric field, which further intensifies the amino protonation of CS molecules in the active layer. At the same time, hydrogen ions gradually migrate to the top electrode during the dynamic process of binding and separating amino and water molecules, and the conductivity of CS molecules near the top electrode is enhanced. When the applied voltage reaches V_SET_, the protated amino groups in the active layer of the CS molecules form conducting filaments, and the upper and lower electrode conduction devices change from the HRS to the LRS. The Al/CS:MWCNTs in MWCNTs/ITO devices provide a large number of oxygen‐containing functional groups for the active layer. In the low‐voltage region, protons jump through functional groups (Figure 2d). MWCNTs increase the proton migration rate, accelerate the formation of conductive filaments in the active layer of the device, and form oxygen vacancy filaments during the switching process of the device.

### 2.2 Sponge Sensor

**Figure 3:**
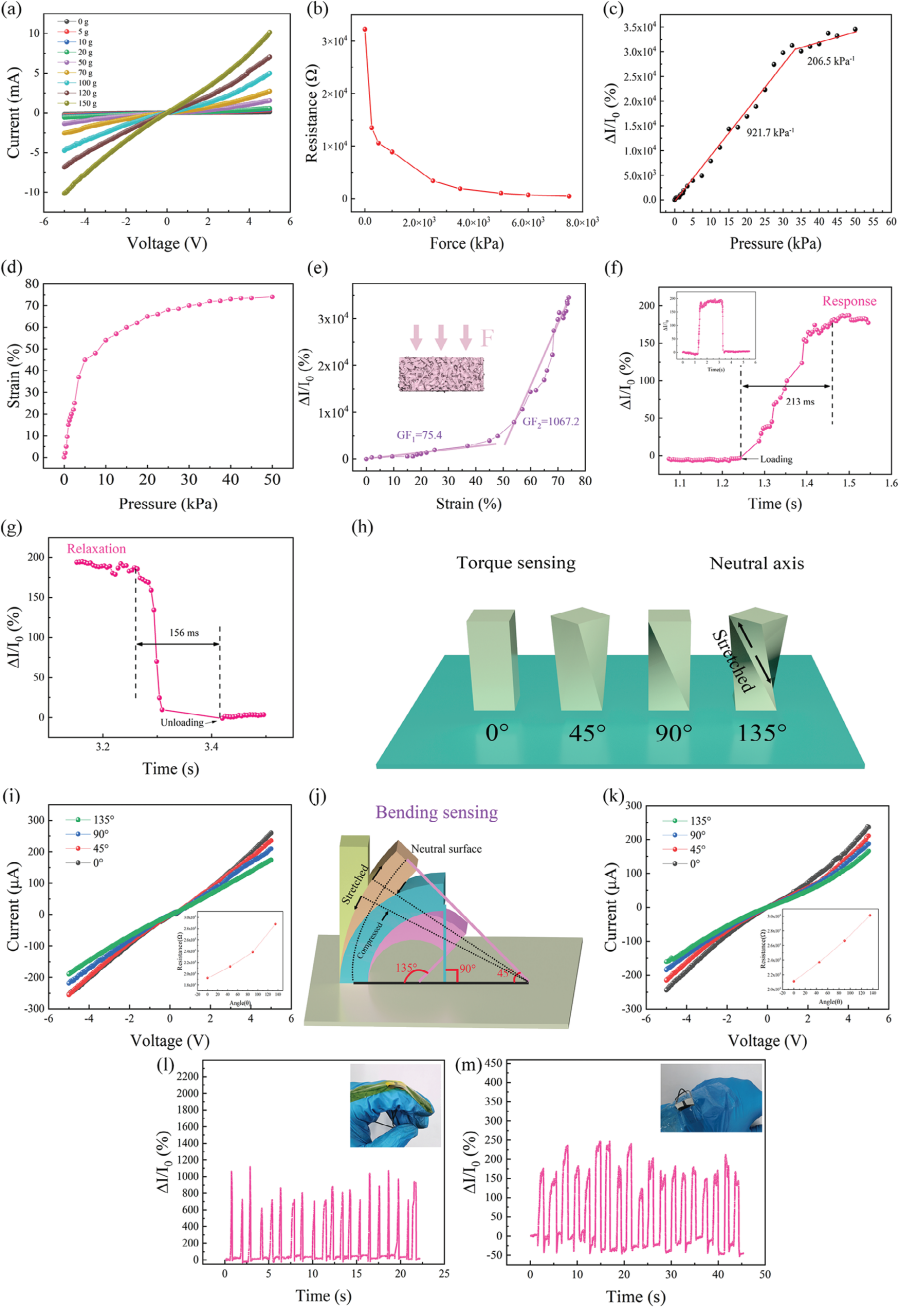
a) *I*–*V* scanning of the sensor under different weights. b) Resistance change. c) Sensitivity test. d) Relationship between device strain and applied pressure. e) Relationship between the sensor strain and current response. f) Response time. g) Recovery time. h) Torque sensing diagram. i) Torsion current response. j) Bending sensing diagram. k) Bending current response. l) Finger bending. m) Wrist bending.

Because sponges are soft materials with hollow structures, they can meet the production needs of flexible pressure sensors, and they can experience obvious strain under pressure. A piezoresistive tactile sensor with excellent performance can be prepared by attaching conductive particles to a 3D hollow structure.^[^ ^38^, ^39^ ^]^ In this study, a CS solution was attached to the hollow structural skeleton of a sponge to form a three‐dimensional CS skeleton containing water molecules and hydrogen ions.^[^ ^37^ ^]^ The sensitivity of the sponge sensor can be further enhanced by doping multiwall carbon nanotubes. Under pressure, the hollow structure of the conductive sponge collapses,^[^ ^40^ ^]^ and the upper and lower contact areas of the skeleton gradually increase, resulting in an increasing number of conductive channels and thus increasing the current response of the device. To study the response of the sensor to pressure, static pressure is applied by placing a weight on the upper surface of the device, and the current response of the device is observed by applying a voltage sweep from −5 to 5 V to the device. As shown in **Figure** **3**a, the slope of the current response curve of the device changes by changing the static pressure applied to the device. With the gradual increase in static pressure, the slope of the curve becomes increasingly larger, indicating that the resistance of the device gradually decreases. The resistance variation of the device under static pressure is plotted in Figure 3b. The figure clearly shows that the resistance of the device changes when pressure is applied at the beginning, and the variation range of the resistance decreases when the pressure gradually increases. The sensitivity of the tactile sensor is shown in Figure 3c. The sensitivity is 921.7 within 0–35 and 206.5 kPa^−1^ within 35–50 kPa. Figure 3d shows the strain of the device under different pressures. It can be seen from the figure that when the pressure is 0–10 kPa, the stress in the device increases obviously with increasing pressure. Under subsequent pressure, the strain of the conductive sponge still increases gradually with increasing pressure. However, due to the small pores in the sponge material at this time, the strain caused by the increase in pressure decreases, and the material finally tends to stabilize and approach the complete compression state. Figure 3e shows the relationship between the device strain and the current response. The sensitivity of the device is GF_1_ = 75.4 in the 0–50% strain range and GF_2_ = 1067.2 in the 50–74% strain range. As shown in Figure 3f,g, a square waveform pressure (0.2 Hz, 2.5 kPa) is applied to the device to test the current change in the device. The response time of the device under pressure was ≈213 ms (Figure 3f), and the recovery time was ≈156 ms (Figure 3g). The overall response time and recovery time of the device are fast.

The sponge sensor will have obvious current response changes under different torsion angles and bending angles. Figure 3h shows a schematic diagram of the device when it is twisted at 0°, 45°, 90°, and 135°. Torsion will cause different current response changes in the device. Whether the device is twisted or bent, the internal force can be decomposed into horizontal and vertical stretching and compression. Figure 3i shows that the larger the twisting angle of the device is, the greater the increase in resistance. When the device is twisted, because the two ends of the device remain fixed, under torsion, the central part of the device is basically not stretched or compressed, and most of the surrounding parts are in a stretched state. With increasing torsion angle, the tensile strength of the device intensifies, resulting in an increase in the resistance and a decrease in the current response. The resistance of the device will also change under different bending angles. As shown in Figure 3j, the bottom end of the device is fixed, and the upper part of the device is driven to bend 0°, 45°, 90°, and 135° to observe the current response of the device. When the bending angle of the device is different, different strain states occur at both ends of the neutral surface of the device, which leads to different resistance changes. In the bending state, tensile strain occurs in the upper part of the neutral plane, and compressive strain occurs in the lower part. Because the tensile strain changes more than the compressive strain, the resistance of the device gradually increases as the bending angle increases (Figure 3k).

Based on the resistance change characteristic of the sensor when strain occurs, finger bending is monitored by the sensor. Figure 3l shows the sensor positioned at the knuckle of the hand to monitor the current response of the device through the wire in real time. The sensor can be stretched and compressed in real time as the finger bends, so the current response changes. Figure 3m shows the changes in the current response of the device tested by placing the sensor on the wrist. As shown in the figure, similar to that in the finger test, the current response of the device also exhibited periodic changes.

### 2.3 Artificial Tactile Sensing Neuron

**Figure 4:**
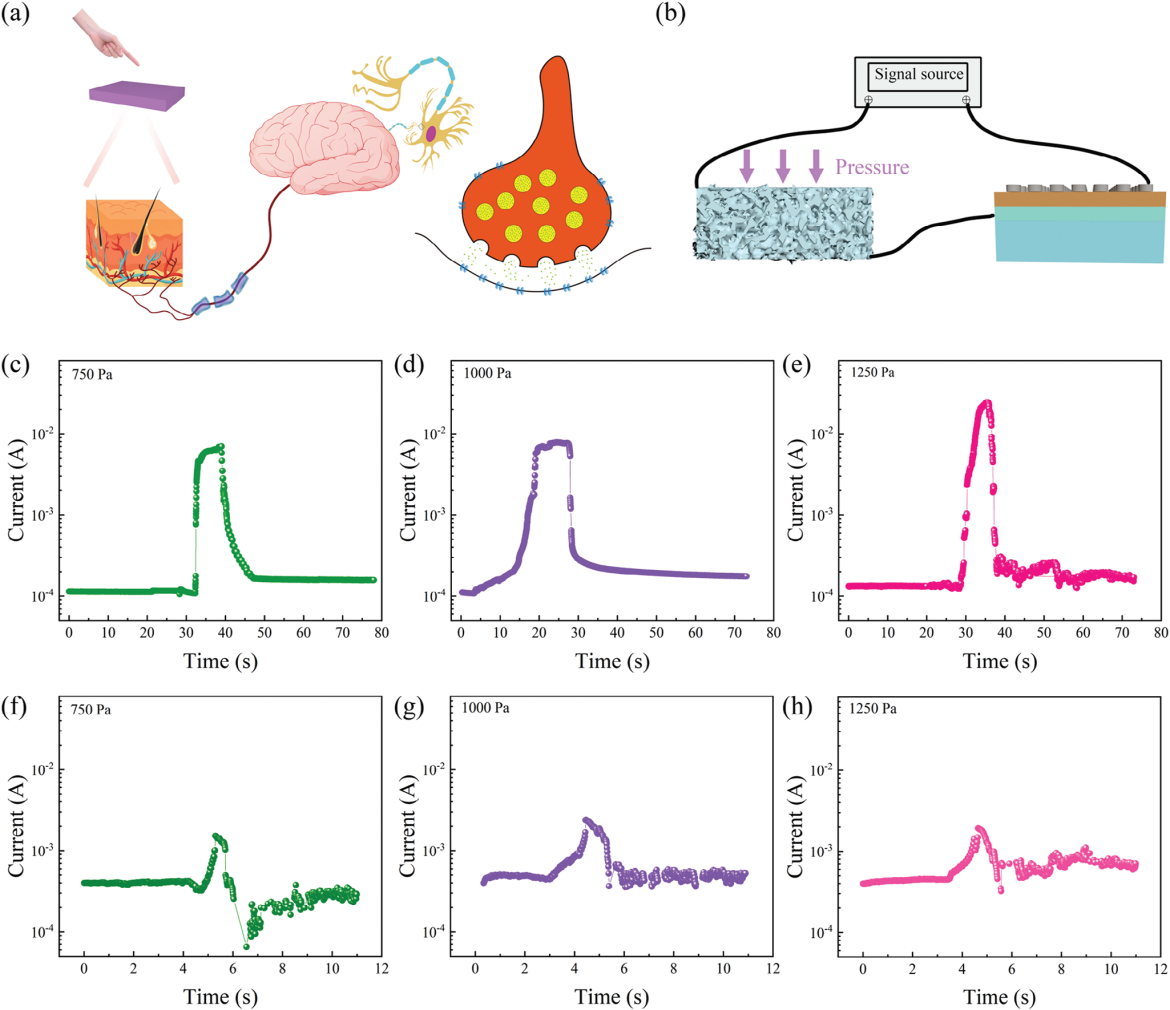
a) Composition of neurons. b) Device connection diagram. c–e) Neurons that store stress information at 750, 1000, and 1250 Pa. f–h) Neurons erase information under stresses of 750, 1000, and 1250 Pa.

Tactile neurons can manipulate the position of a memristor by changing the sensor resistance. **Figure** **4** shows the current response of neurons during the whole process of pressure action and release, and the voltage at both ends of the neurons is 2 V. As shown in Figure **4**a, when 750 Pa stress is applied to the sensor, the neuron output current immediately changes significantly. When the applied pressure was removed, the current response gradually decreased, and the sensor gradually returned to the initial state. The current value finally stabilized at 1.57 × 10^−4^ A. Compared with the initial current response of 1.07 × 10^−4^ A, the overall current response of the neuron after the sensor is released by force is greater. This indicates that the voltage at both ends of the memristor reached the setting condition during the sensor operation, and a state switch from the HRS to the LRS occurred, reducing the overall resistance value. Figure 4d,e shows the current response of neurons under pressures of 1000 and 1250 Pa, respectively, and the current response in the final state is 1.75 × 10^−4^ and 1.82 × 10^−4^ A, respectively. The final current response increases gradually in four tests under different pressures, which corresponds to the multistage storage function of the memristor.

Figure 4f‐h shows the results of the memristor reset test of neur.ons. The reset voltage of the device is ≈2.95 V, and the voltage at both ends of the tactile neuron is changed to 5 V, where the top electrode of the memristor is connected to the positive electrode of the power supply. Figure 4f shows the current response of tactile neurons after 750 Pa is applied to the sensor. Under the action of stress, the current response gradually increases due to the decrease in the sensor resistance. It can be clearly seen in the figure that there is a downward jump in the process of increasing the current response, which also proves that the memristor in the tactile neurons is reset to the HRS. The current response of the memristor gradually stabilizes at 2.88 × 10^−4^ A after the state change. As shown in Figure 4g,h, the current response curves under stresses of 1000 and 1250 Pa all jump downward during the growth process, and the current responses stabilize at ≈5.14 × 10^−4^ A and 6.32 × 10^−4^ A, respectively, after the action of the three stresses.

**Figure 5:**
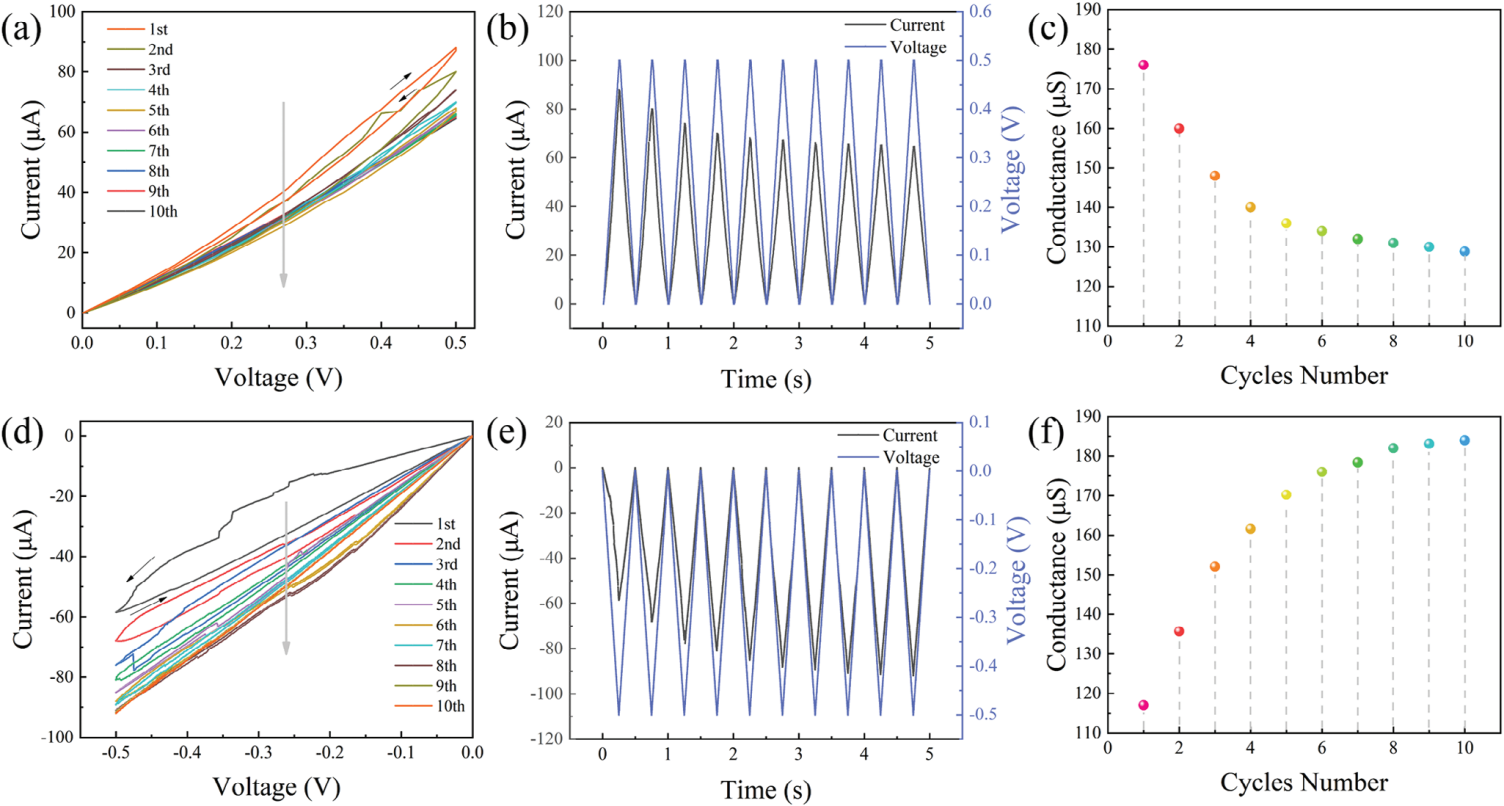
a) Adjustable weight of the positive half region. b) Changes in the positive half. region voltage and current over time. c) Changes in the positive half‐region conductance. d) Adjustable weight of the negative half‐region voltage and current over time. f) Changes in the negative half‐region conductance.

In a neuronal system, the degree of connection between neurons is affected by the synaptic weight. Synapses can dynamically adjust and store information using successive spikes, a process known as synaptic information transfer. To study the weighted harmonic synaptic transmission characteristics of tactile neurons, 10 consecutive positive and negative voltage scans were applied to the devices. As shown in **Figure** **5**a, the current response of the device gradually decreases under continuous positive voltage scanning (0–0.5 V). As shown in Figure 5b, the current response of the device increases gradually under continuous negative voltage scanning (0–−0.5 V).

To observe the current change in the memristor under cyclic scanning, Figure 5c,d shows the relationship between the current response of the device and time under positive and negative scanning voltages, respectively. By counting the conductance under the maximum voltage in the cycle scanning process, it can be clearly observed that the device conductance gradually increases in the negative half region and gradually decreases in the positive half region (Figure 5e,f). This indicates that repeated DC voltage biases can change the conductance of the device. The electrical conductance of neurons can mimic the weight of biological synapses.

**Figure 6:**
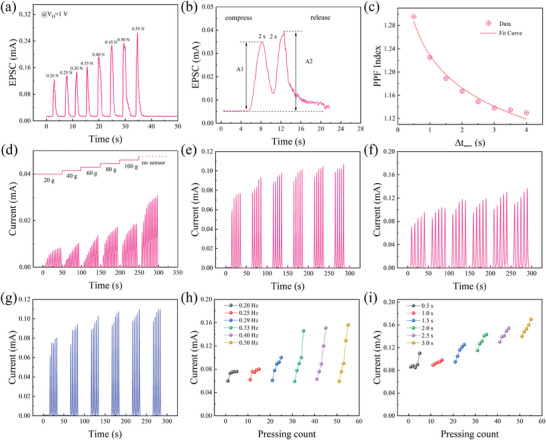
a) Changes in the EPSC under different stresses. b) PPF behavior test. c) Changes in the PPF index with time interval. d) Current response under static pressure of different weights. e) Postsynaptic current under 0.17 Hz stress. f) Postsynaptic current at 0.125 Hz. g) Postsynaptic current at 0.25 Hz. h) By changing the interval of the pulse train, the pressing frequency was increased from 0.20–0.5 Hz. i) The pressing duration was 0.5–3.0 s at 2.5 kPa.

**Figure** **6**a shows the changes in excitatory postsynaptic currents (EPSC) in neurons after different stresses are applied to the sensor. The applied stress increased from 0.20 to 0.55 N, and the duration of each applied force was ≈2 s. The triggered EPSC size increased from 0.09 to 0.26 mA for different stresses. Under the action of greater stress, a greater excitability output signal is generated. The time interval Δt_pre_ is set to 1 s by applying two consecutive stresses of 0.15 N and 1 s on the sensor. The overall current response of neurons showed paired‐pulse facilitation (PPF) behavior. Peak A2 of the second EPSC is slightly greater than peak A1 of the first EPSC (Figure 6b). Figure 6c shows that the PPF index decreases as the time interval Δt_pre_ increases, and the maximum value is 1.29 when Δt_pre_ = 0.50 s in the test. Figure 6d shows the postsynaptic current of the neuron after receiving the voltage signal under different weights. The changes in the current response were consistent across the six test datasets, and all the postsynaptic current peaks were positively correlated with the applied weight. As the number of pulses used by artificial tactile neurons depends on plasticity, the postsynaptic current increases gradually in each group of experiments, and the increase in the current response decreases gradually with increasing pulse excitation time. Electrical pulses can be applied to a memristor by varying the amount of force applied to the tactile sensor.

To investigate the effect of the pulse width of the pressure stimulus on the postsynaptic current, a 1 V voltage was applied to both ends of the neuron. A force of 0.20–0.45 N was applied to the sensor, the pulse width of each force was 2 s, and the interval was 4 s. After five consecutive pressure signals, the postsynaptic current gradually increased (Figure 6e). The width of the force pulse was adjusted to 4 s, and the pulse interval was adjusted to 4 s. The postsynaptic current obtained under the same force also showed a trend toward gradual enhancement (Figure 6f), and an increase in the pulse width led to a greater increase in the current response. To observe the effect of pulse frequency on the postsynaptic current, the force pulse width was set to 2 s, and the interval was set to 2 s. Under the 0.25 Hz force pulse signal, the current response increases slowly (Figure 6g), and a pulse signal with the same amplitude and higher frequency generates a stronger postsynaptic current, which verifies the pulse frequency‐dependent plasticity of artificial tactile neurons. Figure 6h shows the change in the postsynaptic current response of the device under the action of a force with a frequency of 0.2–0.5 Hz and a size of 0.2 N. A force with a higher frequency can enhance the overall current response of the device. Figure 6i shows the current response of the device under a stress of 0.2 N and a frequency of 0.2 Hz. A stress with a longer pulse width generates a stronger postsynaptic current.

## 3 Conclusion

In this paper, a low‐cost and easily fabricated artificial tactile neuron is constructed by integrating a flexible tactile sensor and a CS‐based memristor. The tactile sensor is used as a sensory receptor to convert physical stimuli into electrical signals. The sensitivity of the sensor is 921.7 within 0–30 and 206.5 kPa^−1^ within 30–50 kPa, which can realize the acquisition of tactile information. CS‐based memristors have stable bipolar resistance switching characteristics and can effectively process tactile information. Two kinds of devices are combined to simulate biological sensory neurons and realize the storage and erasure of tactile information. Artificial touch neurons can integrate tactile stimuli related to time; realize the modulation of synaptic weight potentiation/depression, the PPF, spike‐rate‐dependent plasticity and spike‐rate‐dependent plasticity for sensory information processing and learning; and provide a new idea for a new wearable, low‐cost artificial touch system.

## 4 Experimental Section

CS powder (degree of deacetylation > = 95%) was dissolved in 2 wt.% acetic acid aqueous solution at room temperature and stirred continuously for more than 5 h at room temperature. A mixture of 0.4, 1.2, and 3 wt.% MWCNTs and deionized water was prepared. The CS solution was mixed with the dispersion of MWCNTs at three concentrations at a volume ratio of 1:1, and ultrasonication was applied for 30 min. The resulting solution was spin coated on ITO for 5 s at 500 rpm and 40 s at 2000 rpm. The CS substrate doped with MWCNTs was dried for 30 min under vacuum at 60 °C, and an Al electrode was prepared by the thermal evaporation method.

A CS solution with a concentration of 2 mg ml^−1^ was prepared. The cleaned sponge material was soaked in CS solution for 24 h and then dried in a vacuum drying oven at 50 °C. The sponge with CS on the surface was soaked in a 1.2 wt.% MWCNTs solution for 10 min and then dried in a vacuum drying oven. The previous step was repeated three times to obtain a conductive sponge material.

## Conflict of Interest

The authors declare no conflict of interest.

## Author Contributions

L.W. performed conceptualization, funding acquisition, methodology, wrote the original draft, review and edited the draft. P.Z. and Z.G. performed investigation, software, validation, wrote the original draft. D.W. performed conceptualization.

## Supporting information

Supporting Information

## Data Availability

The data that support the findings of this study are available from the corresponding author upon reasonable request.
