# Supplementary material for: Artificial Tactile Sensing Neuron with Tactile Sensing Ability Based on a Chitosan Memristor
Source: Adv Sci (Weinh). 2024 Mar 14;11(19):2308610. doi: 10.1002/advs.202308610 (PMC11109609; doi:10.1002/advs.202308610)
Supplement: Supplementary file 1 — Supporting Information [file ADVS-11-2308610-s001.pdf]

ADVANCED SCIENCE
Open Access

Supporting Information

for *Adv. Sci.*, DOI 10.1002/advs.202308610

Artificial Tactile Sensing Neuron with Tactile Sensing Ability Based on a Chitosan Memristor

Lu Wang, Peng Zhang, Zhiqiang Gao and Dianzhong Wen*

Supporting Information

Artificial tactile sensing neuron with tactile sensing ability based on a chitosan memristor

Lu Wang, Peng Zhang, Zhiqiang Gao and Dianzhong Wen*

L. Wang, P. Zhang, Z. Gao, D. Wen

School of Electronic Engineering, Heilongjiang University, Harbin 150080, China.

E-mail: wanglu@hlju.edu.cn

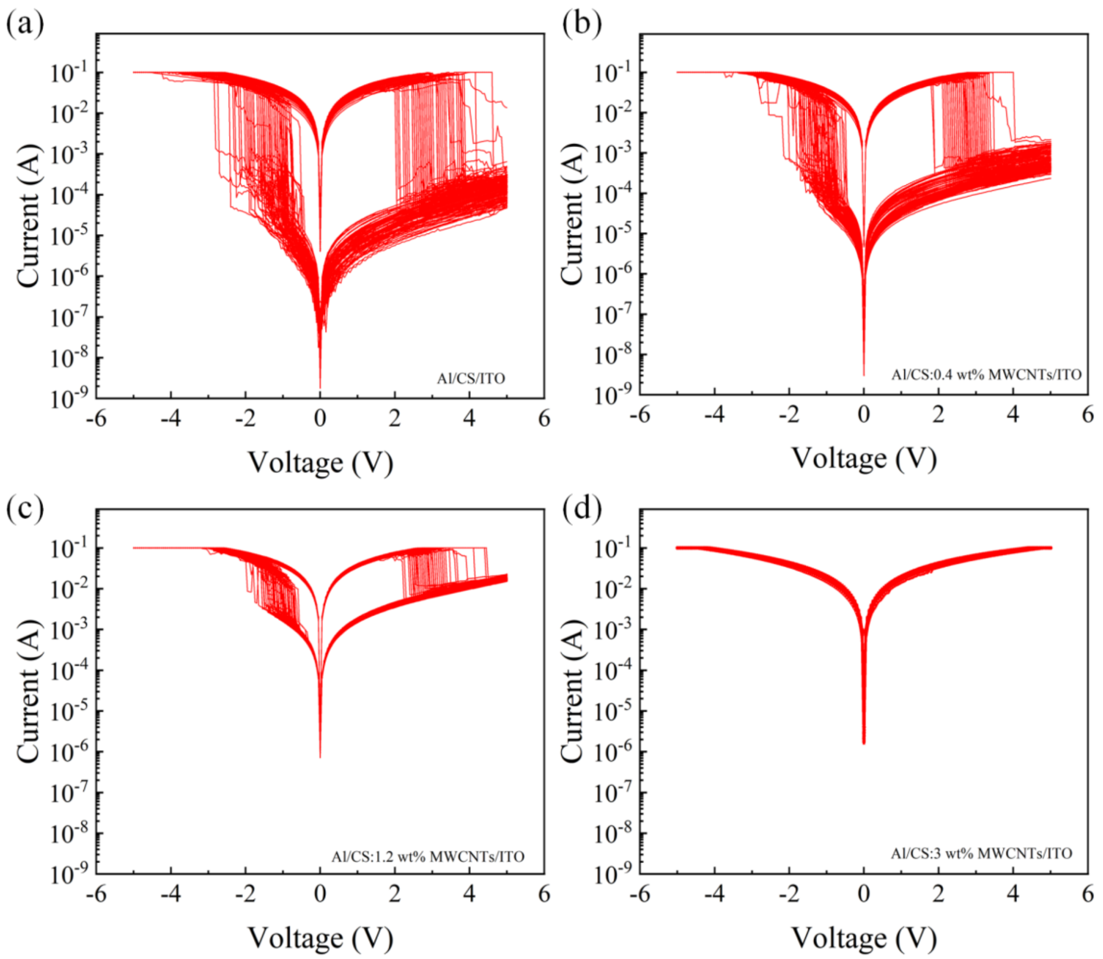

Figure S1. Repeatability testing for the same cell. (a) Al/CS/ITO devices. (b) Al/CS:0.4 wt% MWCNTs/ITO device. (c) Al/CS:1.2 wt% MWCNTs/ITO device. (d) Al/CS:3 wt% MWCNTs/ITO device.
